# Gender Differences in the Prevalence of Chronic Pain and Leisure Time Physical Activity Among US Adults: A NHANES Study

**DOI:** 10.3390/ijerph16060988

**Published:** 2019-03-19

**Authors:** Masataka Umeda, Youngdeok Kim

**Affiliations:** 1 Department of Kinesiology, Health, and Nutrition, the University of Texas at San Antonio, San Antonio, TX 78249, USA; 2 Department of Kinesiology and Sport Management, Texas Tech University, Lubbock, TX 79424, USA; youngdeok.kim@ttu.edu

**Keywords:** gender disparities, chronic pain, physical activity, walking

## Abstract

Gender disparities in chronic pain are well documented in the literature. However, little is known regarding the relationship between physical activity (PA) and gender disparities in chronic pain. This study described gender differences in prevalence of chronic pain and PA, and identified a type of leisure time PA that individuals frequently chose in a nationally representative sample of US adults (*N* = 14,449). Data from the National Health Nutrition Examination Survey 1999–2004 were analyzed. Individuals were categorized into no chronic pain (NCP), localized chronic pain (LCP), and widespread chronic pain (WCP) groups based on responses to a pain questionnaire. A self-report PA questionnaire was used to estimate the time spent in different types of PA. Women showed higher prevalence of LCP and WCP compared to men. Men spent more hours per week for leisure time PA compared to women, but men and women showed similar prevalence of sufficient PA to meet a PA recommendation (≥150 min/week of moderate-to-vigorous intensity PA) across chronic pain categories. However, the prevalence of sufficient PA was substantially higher among men and women with NCP compared to men and women with LCP and WCP. Additionally, both men and women chose walking as the primary type of leisure time PA. Together, gender disparities exist in the prevalence of chronic pain and hours spent for leisure time PA. More research is needed to explore the role of increasing leisure time PA, such as walking, in reducing gender disparities in chronic pain.

## 1. Introduction

It is well known that regular physical activity (PA) plays an important role in improvement and maintenance of health. Over the last decades, several PA recommendations have been published to advocate a certain amount of regular PA that may lead to improvement of health [1,2,3], and a recent PA recommendation specifically encourages US adults to regularly engage in ≥150 min/wk of moderate-to-vigorous intensity aerobic PA (MVPA) for health promotion [1]. Additionally, it is well accepted that regular PA is an essential part of prescribed non-pharmacological treatment for chronic pain [4], and epidemiological evidence shows that regular PA reduces the risk of developing chronic pain [5,6,7]. These observations collectively suggest that the benefits of regular PA may extend into treatment and prevention of chronic pain. However, there is currently a very limited amount of data regarding the prevalence of sufficient PA to meet the PA recommendations among individuals with chronic pain. A recent study conducted with a representative sample of US adults shows the lower prevalence of sufficient PA to meet a PA recommendation, as defined by ≥150 min/wk of leisure-time aerobic PA at light, moderate, and vigorous intensity, among individuals with arthritis when compared to healthy individuals (approximately 45% vs. 55%) [8], suggesting a potential link between sufficient PA to meet the PA recommendation and chronic pain.

It is known that PA behaviors are influenced by multiple factors, including biological, psychological, and social factors [9,10]. Among the biological factors, gender has been consistently found as a PA correlate [9], and epidemiological research conducted with representative samples of US adults show that women are generally less physically active in comparison to men, with lower prevalence of sufficient PA to meet the PA recommendations among women compared to men [11,12]. Given the role of PA in developing chronic pain [5,6,7], the findings suggest the higher risk of chronic pain among women compared to men, and, consistent with the hypothesis, past research generally indicates that women experience chronic pain more frequently compared to men [13,14,15,16,17,18]. Additionally, past studies conducted with representative samples of US adults show that men with chronic pain spend more time engaged in PA compared to women with chronic pain [19], and more men with arthritis meet the PA recommendation compared to women with arthritis [8]. These observations indicate that gender disparities in PA can be observed among chronic pain populations, as well as general populations. However, there is still a paucity of evidence regarding gender disparities in the prevalence of chronic pain and PA, highlighting a need of more research delineating gender differences in chronic pain and PA. Therefore, the purpose of this study was to describe gender differences in the prevalence of chronic pain and PA, and examine gender differences in the preferred types of leisure time PA among US adults using a nationally representative sample of noninstitutionalized adults.

## 2. Methods

### 2.1. Survey Data and Study Population

Data from the continuous National Health and Nutrition Examination Survey (NHANES) were used for this study. The NHANES is an ongoing cross-sectional survey conducted by the National Center for Health Statistics in 2-year cycles to monitor the health and nutritional status of the US population. The data consist of a nationally representative sample of the US civilian noninstitutionalized population selected by a complex, multistage probability sampling design with oversampling of minority population groups such as young children, older adults, and pregnant women. The face-to-face household interview as well as physical examinations at a mobile examination center were conducted to collect comprehensive data on health, medical, and nutrition from the participants. The NHANES protocols were approved by the research ethic review board at the National Center for Health Statistics, and the details of NHANES protocols can be found elsewhere (https://www.cdc.gov/nchs/nhanes/index.htm). For this study, the combined NHANES from the three consecutive cycles (1999–2000, 2001–2002, and 2003–2004), where the personal interview data on pain conditions are available, were analyzed. Of the 31,126 in the sample, we limited our analysis to adults aged 20 years or older after exclusion of women with a positive pregnancy test (*n* = 833), resulting in a final sample size of 14,449 adults. The pregnant women were excluded from analysis in this study to minimize the potential confounding effects of pregnancy on the parameter estimates of PA as the literature shows that women experience unique physical/biological/psychological changes during the course of pregnancy that alter their normal behavioral patterns, including PA [20].

### 2.2. Chronic Pain Assessment

Chronic pain was assessed based on the responses to the miscellaneous pain questionnaire collected during the household interview. Participants were asked if they had a problem with pain that lasted more than 24 h during the past month. If the answer was “yes”, follow-up questions about the duration of symptom (<1 month, 1 to <3 months, 3 to <12 months, and ≥12 months) and which body regions were affected were asked using a pictorial manikin drawn on the hand card. For this study, chronic pain was defined as pain that lasted at least 3 months or more according to the American College of Rheumatology (ACR) [21]. Additional ACR criteria were used to determine widespread chronic pain (WCP), which is defined as pain problems that lasted at least 3 or more months on both sides of the body, above and below the waist, and at one or more axial locations (spine, chest, upper or lower back). Based on these criteria, participants who reported no pain problem during the past month and those who had pain problems less than three months in duration were categorized as no chronic pain (NCP) group. Participants who had chronic pain problems, but did not meet the WCP criteria were categorized as localized chronic pain (LCP) group, and those who met all of ACR criteria were classified as WCP group. We categorized the participants into the three pain groups based on a hypothesis that WCP may affect PA behaviors more than LCP due to pain that results from multiple sources across body.

### 2.3. Self-Reported Physical Activity

During the household interview, self-reported PA data were collected. Participants were asked to report their PA behaviors over the past 30 days. Transportation-related activities including walking or bicycling for commuting and household activities that require moderate or greater physical effort were asked. The frequency and duration of those activities were used to calculate weekly estimates. Separate questions were asked to determine whether they had engaged in moderate and vigorous-intensity activities during their leisure time over the past 30 days, where moderate and vigorous-intensity activities were defined as activities that cause light sweating or a slight to moderate increase in breathing, and activities that cause heavy sweating or large increases in breathing, respectively. The example activities given for each intensity were bicycling for pleasure, brisk walking, dancing, or golf for moderate-intensity activity and fast bicycling, lap swimming, running, or aerobics classes for vigorous-intensity activity. If they answered “yes”, follow-up questions were asked about types, frequency, and duration of activity. The types of activity reported were recorded into forty-eight activity categories including one “other” category based on the Compendium of Physical Activities that provides metabolic equivalent values for each activity category at different intensity levels [22]. For this study, by following the works of Evenson et al. [23,24], the activity categories were further grouped into 10 activity groups (i.e., home maintenance, indoor aerobic conditioning activities, jogging, water activities, recreational activities, stretching, strengthening, team sports, walking, and others) in addition to coding whether they were aerobic activity or not. The participants were categorized into two groups based on the 2008 PA guidelines: (1) those who did not meet current PA recommendations (≥150 min of moderate-intensity aerobic activity, ≥75 min of vigorous-intensity activity, or equivalent combination of moderate and vigorous-intensity activities); and (2) those who meet the recommendation. This categorization was based on both the total time spent in PA behaviors including transportation, household, and leisure activities [25] and leisure time aerobic activity only [24], where one additional category of “no leisure MVPA” was created for the latter approach.

### 2.4. Other Variables

Demographic characteristics of the participants, including age, gender, race/ethnicity, education level, marital status, and annual household income, were obtained. Information on chronic medical conditions was collected by asking if a doctor or other health professional ever told them that they have high blood pressure, diabetes, asthma, arthritis, heart disease, and/or osteoporosis. In addition, body mass index (kg/m^2^) was calculated using the height (cm) and weight (kg).

### 2.5. Statistical Analysis

Descriptive statistics of the participant’s characteristics were calculated. Differences in participant’s characteristics by chronic pain categories were examined using the Rao-Scott chi-square test of independence. Weekly time spent in PA (hours/week) and the percentage of time spent in PA across different types of leisure activity groups were estimated for each chronic pain group by gender. A general linear model was used to estimate the parameters as well as the linear trends in PA outcome variables by chronic pain categories after controlling for study covariates including age, group, race/ethnicity, education level, marital status, weight status, and chronic health conditions. Follow-up Tukey’s pairwise comparisons were conducted between chronic pain categories and between chronic pain categories by gender. The 6-year sampling weights were applied to all analyses using the SAS SURVEY procedures in order to account for the complex sampling design of the NHANES such as oversampling, survey non-response, and post-stratification, and to produce population-based estimates. A statistical significance was considered at *P* ≤ 0.05 unless otherwise specified.

## 3. Results

### 3.1. Prevalence of Chronic Pain and the Participants’ Demographic Data

Table 1 presents the weighted national prevalence of chronic pain by demographic characteristics of the US adults. All demographic variables were significantly associated with chronic pain based on a Rao-Scott *x*^2^ test of independence (*P*’s < 0.05). The results indicated that women showed the higher prevalence of LCP (56.39%; SE = 1.72) and WCP (59.73%; SE = 3.21) compared to men. Further, more adults with WCP were obese (45.57%; SE = 3.21), with a variety of comorbid chronic health conditions, compared to adults with NCP and LCP.

### 3.2. Descriptive Data on the Participants’ Physical Activity

Table 2 presents the descriptive data about self-reported PA by chronic pain status and gender. The results generally indicated a linear, decreasing trend of hours per week for transportation-related and leisure time PAs across chronic pain categories, such that adults with NCP spent more hours per week for transportation-related and leisure time PAs compared to adults with LCP and WCP, and adults with LCP spent more hours per week for leisure time PA compared to adults with WCP (*P*’s for trends <0.001). There was an increase in time spent in household activities across chronic pain categories; yet, the trend was not statistically significant (*P* = 0.396). When data were further stratified by gender, as well as chronic pain status, the results generally showed that men spent more hours per week for leisure time PA compared to women across chronic pain categories; however, statistically significant gender differences were not as consistently observed in adults with LCP and WCP as adults with NCP.

### 3.3. Descriptive Data on the Types of Leisure Time Physical Activity

Table 3 shows the types of leisure time PA and the proportion of hours they spent for each activity. Results indicated that walking was the most common leisure time PA among adults, regardless of chronic pain status, with the largest proportion of time (ranges between 37.00–39.17%), followed by recreational activities (15.32–20.89%) and indoor aerobic conditioning activities (12.30–14.65%). On the other hand, jogging was the leisure time PA that adults with LCP (1.65%; SE = 0.57) and WCP (1.06%; SE = 1.22) spent the smallest proportion of time for among all activities (except for others). Likewise, adults with NCP spent limited time engaged in jogging, but spent a larger proportion of time jogging (3.53%; SE = 0.58) compared to adults with LCP and WCP. In general, adults did not spend much time stretching, but adults with WCP spent a larger proportion of time stretching (7.16%; SE = 1.74) compared to those with NCP (2.39%; SE = 0.74) and LCP (2.36%; SE = 0.78). When further considered with gender, as well as chronic pain status, results showed that men spent a major proportion of their time engaged in recreational activities and walking (approximately 20–30% for each) across chronic pain categories. On the other hand, women spent the largest proportion of time engaged in walking (approximately 45%) across chronic pain categories.

### 3.4. Prevalence of Meeting the PA Recommendation

Data on the prevalence of meeting the MVPA recommendation are presented in Table 4. The results indicated that adults with LCP and WCP (58.85%; SE = 2.58) showed a higher prevalence of no leisure time MVPA (44.27% and 58.85% for those with LCP and WCP, respectively) and lower prevalence of sufficient leisure time MVPA (24.24% and 14.23% for those with LCP and WCP, respectively) compared to adults with NCP. When the data were further stratified by gender, as well as chronic pain status, women with NCP showed the higher prevalence of no leisure time MVPA and lower prevalence of insufficient leisure time MVPA compared to men with NCP. However, no significant gender difference was observed in the prevalence of sufficient leisure time MVPA in adults with NCP. In contrast, among adults with LCP and WCP, there were no significant gender differences observed in the prevalence of sufficient leisure time MVPA, insufficient leisure time MVPA, and no leisure time MVPA (*P*’s > 0.05). Although gender differences were not observed in the prevalence of sufficient leisure time MVPA across chronic pain categories, the results showed that the prevalence of sufficient leisure time MVPA was substantially higher among men and women with NCP compared to men and women with LCP and WCP. Similar patterns were observed when categorizing the participants using total MVPA minutes, but a higher prevalence of sufficient MVPA (≈20%) was estimated across chronic pain categories and gender.

## 4. Discussion

The present study aimed to describe gender differences in the prevalence of chronic pain and PA among US adults, and identify the types of leisure time PA that the individuals frequently engaged in during the leisure time. Results indicated that women showed higher prevalence of LCP and WCP compared to men, and men engaged in more leisure time PA compared to women. However, men and women showed similar prevalence of sufficient MVPA across chronic pain categories, but the prevalence of sufficient MVPA was substantially higher among men and women with NCP compared to men and women with LCP and WCP. Closer inspection of the data further revealed that women across chronic pain categories spent more time walking as their primary choice of leisure time PA compared to other activities, whereas men spent more time engaged in recreational activities and walking as their primary choices of leisure time PA compared to other activities. Together, results from this study described the presence of gender disparities in the prevalence of chronic pain and time spent for leisure time PA in the US, whereas the prevalence of sufficient PA to meet the PA recommendation was comparable between men and women across chronic pain categories.

The higher prevalence of chronic pain among women compared to men was consistent with past research [13,14,15,16,17,18]. It is still unclear what places women at a greater risk of chronic pain compared to men. However, the observations regrading women’s elevated risk of chronic pain may be in agreement with results from laboratory pain research, where women are generally found to be more sensitive to experimental pain stimuli when compared to men [13,26]. Additionally, there has been a growing interest in a laboratory pain test that examines descending pain inhibitory processing, termed as conditioned pain modulation (CPM), and research generally demonstrates that CPM serves as a risk factor of developing chronic pain, such that individuals with smaller CPM tend to be at a greater risk of chronic pain compared to individuals with larger CPM [27,28,29]. To be consistent with these observations, there is some evidence in the literature that women exhibit smaller CPM compared to men [13,28,30,31]. Together, these observations suggest that women’s less efficient pain inhibitory processing and increased sensitivity to pain may be, at least partially, responsible for the elevated risk of chronic pain compared to men. However, identifying a single mechanism to explain gender differences in chronic pain is beyond the scope of the present study, and it has been suggested that multiple factors may be involved in gender differences in pain, such as endocrinological influence, gender roles, cognitive and affective states, body size and functional capacity, occupational options [13,14]. More research is needed to better understand the mechanisms that may explain the observed gender differences in chronic pain.

The present study reported the presence of gender disparities in chronic pain and hours spent for leisure time PA. The present study did not address the causal role of PA in reducing chronic pain; therefore, it is currently unclear whether women’s reduced leisure time PA plays a role in gender disparities in chronic pain. However, the findings could potentially imply a hypothetical role of PA in reducing gender disparities in chronic pain, given that past research indicates that regular PA may help treat [4] and prevent chronic pain [5,6,7]. Although the underlying mechanisms of the PA effect on chronic pain are still poorly understood, growing evidence from experimental studies demonstrates that physically active individuals, such as endurance athletes, typically show reduced sensitivity to experimental pain stimuli compared to normally active healthy individuals [32,33,34]. Furthermore, there is some evidence that those who are sufficiently physically active to meet the PA recommendations show reduced sensitivity to experimental pain stimuli [35] and greater CPM when compared to those who are not sufficiently active [36]. Additionally, results from exercise intervention studies show that individuals who have completed an aerobic exercise intervention exhibit decreased sensitivity to experimental pain stimuli compared to before the intervention [37,38]. Together, these observations collectively suggest that regular engagement in PA may be beneficial for treatment and prevention of chronic pain via improvement of descending pain inhibitory processing, as well as pain sensitivity, and meeting PA recommendations may be a realistic, evidence-based dose of PA that may lead to prevention of chronic pain. However, more research is needed to identify the role of PA in reducing gender disparities in chronic pain.

While gender differences in leisure time PA were observed in the present study, the results showed that both men and women chose walking as their primary form of leisure time PA. We speculate that this is because walking does not necessarily require individuals to have special equipment or devices, and waking is a type of exercise that has been introduced in the past PA recommendations [1,2,3]. Individuals can readily engage in walking in their own neighborhood; however, it is important to note that walking may be a type of leisure time PA that is highly influenced by social and environmental factors. For example, walking in the neighborhood requires some social and environmental infrastructures developed in the area, including safe community, adequate lighting, presence of sidewalks, reduced/controlled traffic, and so on [9,10]. It is beyond the scope of this study to examine the role of leisure time PA in chronic pain prevalence, but it is likely that promotion of leisure time PA, such as walking, requires approaches to modify social and environmental factors of PA.

Absence of gender differences in the prevalence of sufficient MVPA across chronic pain categories was not in agreement with a previous study reporting that more men with arthritis met the PA recommendation compared to women with arthritis [8]. It is currently unclear what is responsible for these inconsistent findings across the two studies; however, methodological differences between the studies may potentially be the explanation. For example, the present study categorized individuals with chronic pain into LCP and WCP, whereas the previous study limited their chronic pain population to individuals with arthritis. Additionally, location of LCP may also be a source of discrepancy between the studies since arthritis may differentially affect specific joints of the body compared to the potential sources of musculoskeletal pain. Furthermore, sufficient PA to meet the PA recommendation was defined differently across the studies. These methodological differences may be, at least partially, responsible for the differences in the findings. More research is needed in the future to examine the role of sufficient PA to meet the PA recommendation in the well-documented gender disparities in chronic pain.

There are limitations in the present study. First, the present study was conducted with archival data collected in a cross-sectional design, and the use of cross-sectional data limit the predictive validity of the findings. Second, because the NHANES discontinued to ask about the ‘miscellaneous pain’ variables since 2005–2006 cycle, we had to use the data in previous cycles, which limits the generalizability of our findings to the current US population. Although the national prevalence of PA has not been drastically changed over the past decade [39,40], the caution is still needed when interpreting the reported prevalence data from this study. In addition, there are currently few national surveys collecting the variables that can be used to describe the symptoms of chronic widespread pain, as well as PA. Given the public health impact of chronic pain, we suggest that continuous efforts be made to better understand causes, prevention, and treatment of chronic pain at national level. Third, we used subjective PA data for the present study, rather than objective PA data, to take advantage of larger sample size of the subjective PA data. However, the use of subjective PA data could be a methodological limitation of the present study. Fourth, we categorized individuals into the NCP, LCP, and WCP groups, and this approach resulted in creating large disparities in sample size across the three groups due to the low prevalence of chronic pain in comparison to the prevalence of no chronic pain. Consequently, it is possible that the sample size differences across the groups may have affected results from statistical analyses. Lastly, PA was categorized in the present study based on the 2008 Physical Activity Guidelines for Americans. However, the guidelines are scheduled to be updated in the near future [41]. Therefore, investigators of future research should consider categorizing PA using the updated guidelines. Together, these limitations require caution in interpreting the results from the present study, and should be considered for the future studies.

## 5. Conclusions

The present study reported that more women experienced chronic pain compared to men, and men engaged in more leisure time PA compared to women. However, no gender differences were observed in prevalence of sufficient PA to meet the PA recommendation across chronic pain categories, but the prevalence of the sufficient PA was substantially higher among pain-free men and women compared to men and women with chronic pain. Furthermore, men and women chose walking as their primary type of exercise during the leisure time. Taken together, this study demonstrates the presence of gender disparities in chronic pain and leisure time PA in the US. More research is needed to explore the potential role of increasing leisure time PA, such as walking, in attenuating gender disparities in chronic pain in the US.

## Figures and Tables

**Table 1:** Prevalence of Chronic Pain Conditions in the US Adults (NHANES 1999–2004).

	No Chronic Pain	Localized Chronic Pain	Widespread Chronic Pain	*P*-value ^a^
Total				
Unweighted (*n*)	12,387	1608	504	
Weighted, % (SE)	84.52 (0.65)	11.85 (0.51)	3.62 (0.24)	
Gender, % (SE)				<0.001
Men	49.92 (0.50)	43.61 (1.72)	40.27 (3.21)	
Women	50.08 (0.50)	56.39 (1.72)	59.73 (3.21)	
Age groups, % (SE)				<0.001
Young adults	42.00 (0.86)	31.21 (1.51)	27.09 (3.25)	
Middle aged adults	36.22 (0.70)	44.28 (1.55)	52.66 (3.39)	
Older adults	21.78 (0.60)	24.51 (1.14)	20.25 (1.84)	
Race/ethnicity, % (SE)				<0.001
Non-Hispanic White	70.83 (1.64)	78.70 (1.99)	77.92 (2.63)	
Non-Hispanic Black	11.38 (1.03)	9.15 (0.98)	10.49 (1.48)	
Other Hispanic/Races	17.79 (1.62)	12.15 (1.85)	11.59 (2.35)	
Education level, % (SE)				<0.001
<High school	20.30 (0.71)	21.12 (1.21)	28.49 (2.38)	
High school or equivalent	25.66 (0.79)	29.61 (1.39)	27.69 (2.66)	
Some college or AA	28.94 (0.74)	31.42 (1.32)	31.23 (2.38)	
College grade or above	25.10 (1.10)	17.84 (1.13)	12.59 (2.07)	
Marital status, % (SE)				0.015
Married or living with partner	62.36 (0.9)	66.77 (1.78)	65.12 (2.49)	
Others	37.64 (0.9)	33.23 (1.78)	35.88 (2.49)	
Annual household income, % (SE)				<0.001
<$35k	37.88 (1.17)	41.07 (2.18)	52.00 (2.97)	
$35k–<$65k	28.39 (0.81)	31.31 (1.49)	28.63 (2.78)	
≥$65k	33.73 (1.49)	27.62 (1.97)	19.37 (2.34)	
Weight status ^b^, % (SE)				<0.001
Normal	35.98 (0.74)	30.49 (1.25)	25.40 (2.77)	
Overweight	34.69 (0.68)	33.81 (1.37)	29.03 (2.68)	
Obese	29.33 (0.80)	35.70 (1.34)	45.57 (3.21)	
Health conditions, % (SE)				
High BP	25.27 (0.68)	34.85 (1.66)	38.42 (2.38)	<0.001
Diabetes	6.17 (0.29)	9.88 (0.91)	13.84 (1.56)	<0.001
Asthma	10.95 (0.38)	16.17 (1.12)	20.67 (2.45)	<0.001
Arthritis	17.70 (0.53)	41.48 (1.65)	65.71 (2.61)	<0.001
Heart disease	2.95 (0.22)	5.88 (0.49)	8.18 (1.35)	<0.001
Osteoporosis	3.98 (0.26)	7.66 (0.82)	18.11 (2.19)	<0.001

^a^*P*-value for a Rao-Scott Chi-square test of independence; ^b^ weight status was determined based on the body mass index (kg/m^2^).

**Table 2:** Summary of Weekly Time Spent in Physical Activity Across Chronic Pain Categories (NHANES 1999–2004).

	No Chronic Pain		Localized Chronic Pain		Widespread Chronic Pain		*P*-value ^a^
	Men	Women	Men	Women	Men	Women	
Transportation activity	0.99 (0.22) ^‡^		0.76 (0.20)		0.46 (0.19)		0.022
Household activity	2.08 (0.29)		2.31 (0.30)		2.48 (0.47)		0.396
Total LPA	3.23 (0.21) ^‡^		2.95 (0.27) ^‡^		2.01 (0.30)		<0.001
Moderate LPA	1.89 (0.15) ^‡^		1.82 (0.20) ^‡^		1.27 (0.21)		0.003
Vigorous LPA	1.34 (0.15) ^‡^		1.13 (0.17) ^‡^		0.73 (0.21)		0.035
Total aerobic LPA	2.12 (0.15) ^†‡^		1.92 (0.17) ^‡^		1.37 (0.24)		0.008
Moderate LPA	1.15 (0.09) ^‡^		1.07 (0.14) ^‡^		0.72 (0.15)		0.004
Vigorous LPA	0.98 (0.10)		0.84 (0.12)		0.64 (0.18)		0.088
*(By gender)*							
*Transportation activity*	*1.17 (0.22)*	*0.80 (0.25)*	*1.00 (0.25)*	*0.52 (0.20)*	*0.52 (0.21)*	*0.38 (0.22)*	
*Household activity*	2.38 (0.32) *	*1.78 (0.28)*	*2.43 (0.37)*	*2.19 (0.36)*	*2.37 (0.61)*	*2.58 (0.70)*	
*Total LPA*	3.85 (0.25) *	*2.61 (0.20)*	3.78 (0.38) *	*2.24 (0.23)*	*2.39 (0.41)*	*1.63 (0.31)*	
*Moderate LPA*	2.11 (0.16) *	*1.67 (0.15)*	2.08 (0.32) *	*1.57 (0.18)*	*1.21 (0.26)*	*1.32 (0.26)*	
*Vigorous LPA*	1.75 (0.16) *	*0.93 (0.14)*	1.60 (0.23) *	*0.67 (0.14)*	1.18 (0.35) *	*0.30 (0.15)*	
*Total aerobic LPA*	*2.18 (0.15)*	*2.07 (0.16)*	*2.12 (0.25)*	*1.70 (0.15)*	*1.49 (0.33)*	*1.23 (0.23)*	
*Moderate LPA*	1.06 (0.10) *	*1.23 (0.10)*	*1.03 (0.21)*	*1.12 (0.12)*	*0.61 (0.17)*	*0.85 (0.19)*	
*Vigorous LPA*	1.11 (0.10) *	*0.83 (0.12)*	1.09 (0.17) *	*0.59 (0.09)*	*0.89 (0.31)*	*0.39 (0.11)*	

Values are average hours per week (SE) estimated from general linear model controlling for study covariates including age, group, race/ethnicity, education level, marital status, weight status, and chronic health conditions. The gender-specific estimates for each pain condition were italicized; LPA = leisure-time physical activity; ^a^ *P*-value from a linear trend unless otherwise specified; ^b^ *P*-value from a Rao-Scott Chi-square test of independence; ^†^ significantly different with the Localized chronic pain; ^‡^ significantly different with the widespread chronic pain; * significantly different with females (*P* < 0.05).

**Table 3:** Types of Leisure Time Physical Activity Across Chronic Pain Categories (NHANES 1999–2004).

	No Chronic Pain		Localized Chronic Pain		Widespread Chronic Pain		*P*-value ^a^
	Men	Women	Men	Women	Men	Women	
Home maintenance	6.73 (1.72)		8.31 (1.04)		8.99 (2.43)		0.560
Indoor aerobic activities	12.30 (1.58)		12.43 (1.96)		14.65 (1.96)		0.506
Jogging	3.53 (0.58) ^†‡^		1.65 (0.57)		1.06 (1.22)		0.048
Water activities	6.80 (1.46)		8.42 (1.64)		5.40 (1.76)		0.331
Recreational activities	19.47 (1.76)		20.89 (2.02)		15.32 (3.81)		0.232
Stretching	2.39 (0.74) ^‡^		2.36 (0.78) ^‡^		7.16 (1.74)		0.004
Strengthening	5.00 (0.71)		4.19 (0.95)		6.60 (1.90)		0.362
Team sports	4.25 (0.72)		3.13 (1.05)		4.79 (2.36)		0.808
Walking	39.17 (2.18)		37.98 (2.57)		37.00 (4.62)		0.631
Others	0.37 (0.23)		0.64 (0.47)		0.00 (0.25) ^c^		0.060
*(By gender)*							
*Home maintenance*	7.37 (1.69) *	*6.09 (1.80)*	*8.49 (2.71)*	*8.14 (2.32)*	*10.10 (4.12)*	*6.14 (3.28)*	
*Indoor aerobic activities*	8.12 (1.52) *	*16.49 (1.71)*	9.89 (2.34) *	*14.98 (2.12)*	10.70 (4.02) *	*18.60 (4.38)*	
*Jogging*	4.78 (0.74) *	*2.28 (0.61)*	3.51 (0.86) *	*0.00 (0.61) ^c^*	*2.54 (2.24)*	*0.00 (0.68) ^c^*	
*Water activities*	7.95 (1.51) *	*5.65 (1.47)*	10.55 (2.10) *	*6.30 (1.59)*	*3.54 (1.47)*	*7.25 (2.83)*	
*Recreational activities*	23.03 (1.80) *	*15.90 (1.91)*	24.85 (2.25) *	*16.93 (2.34)*	*21.71 (5.34)*	*8.93 (5.15)*	
*Stretching*	1.37 (0.76) *	*3.40 (0.76)*	*1.79 (0.98)*	*2.92 (0.83)*	*4.60 (2.88)*	*9.71 (1.97)*	
*Strengthening*	7.44 (0.79) *	*2.56 (0.71)*	6.09 (1.32) *	*2.28 (0.98)*	*9.81 (3.83)*	*3.40 (1.51)*	
*Team sports*	8.59 (0.81) *	*0.00 (0.75) ^c^*	5.86 (1.54) *	*0.41 (0.99)*	*7.93 (3.78)*	*1.66 (2.09)*	
*Walking*	31.04 (2.29) *	*47.30 (2.22)*	28.40 (2.89) *	*47.56 (3.08)*	29.31 (4.92) *	*44.70 (6.21)*	
*Others*	*0.31 (0.26)*	*0.42 (0.24)*	*0.58 (0.63)*	*0.70 (0.63)*	*0.00 (0.22) ^c^*	*0.04 (0.36)*	

Values are % of average hours per week estimated from general linear model controlling for study covariates including age group, race/ethnicity, education level, marital status, weight status, and chronic health conditions. The gender-specific estimates for each pain condition were italicized; ^a^ *P*-value from a linear trend unless otherwise specified; ^†^ significantly different with the Localized chronic pain; ^‡^ significantly different with the widespread chronic pain; * significantly different with females (*P* < 0.05).

**Table 4:** Prevalence of Meeting Physical Activity Recommendation (NHANES 1999–2004).

	No Chronic Pain		Localized Chronic Pain		Widespread Chronic Pain		*P*-value ^a^
	Men	Women	Men	Women	Men	Women	
Leisure MVPA ^b^							<0.001
No Leisure MVPA	35.99 (0.84)		44.27 (1.61)		58.85 (2.58)		
Insufficient (<150 min/wk)	32.43 (0.59)		31.49 (1.59)		26.92 (1.97)		
Sufficient (≥150 min/wk)	31.58 (0.85)		24.24 (1.95)		14.23 (1.67)		
*(By gender)*							
*No Leisure MVPA*	*33.27 (0.92)*	*38.70 (0.99)*	*43.92 (2.24)*	*44.54 (1.97)*	*57.45 (4.59)*	*59.79 (3.64)*	
*Insufficient (<150 min/wk)*	*35.00 (0.76)*	*29.88 (0.79)*	*31.39 (2.31)*	*31.50 (1.80)*	*29.62 (4.15)*	*25.10 (2.81)*	
*Sufficient (≥150 min/wk)*	*31.73 (0.89)*	*31.42 (1.09)*	*24.59 (2.34)*	*23.96 (2.07)*	*12.93 (2.31)*	*15.11 (2.23)*	
*P-value ^a^*	*0.769*		*0.098*		*0.489*		
Total MVPA ^c^							
Insufficient (<150 min/wk)	46.40 (0.91)		50.69 (1.73)		62.69 (3.21)		<0.001
Sufficient (≥150 min/wk)	53.60 (0.91)		49.31 (1.73)		37.31 (3.21)		
*(By gender)*							
*Insufficient (<150 min/wk)*	*44.63 (1.02)*	*48.16 (1.10)*	*48.07 (1.94)*	*52.71 (2.38)*	*66.10 (3.90)*	*60.39 (3.57)*	
*Sufficient (≥150 min/wk)*	*55.37 (1.02)*	*51.84 (1.10)*	*51.93 (1.94)*	*47.29 (2.38)*	*33.90 (3.90)*	*39.61 (3.57)*	
*P-value ^a^*	*0.001*		*0.091*		*0.138*		

Values are presented as the weighted % (SE) of US adult population. The gender-specific estimates for each pain condition were italicized; ^a^ *P*-value from a Rao-Scott Chi-square test of independence; ^b^ categorization was based on the time spent in leisure time aerobic activity only (Evenson et al., 2014); ^c^ categorization was based on the total time spent in transportation, household, and leisure time aerobic activities (Tucker et al., 2011).
